# Typology and Characteristics of COVID-19 Preventive Measures Implementation

**DOI:** 10.3390/ijerph191912194

**Published:** 2022-09-26

**Authors:** Motoko Kosugi

**Affiliations:** College of Engineering, Academic Institute, Shizuoka University, Hamamatsu 432-8561, Japan; kosugi.motoko@shizuoka.ac.jp

**Keywords:** COVID-19, preventive measures, risk perception, affect, questionnaire survey

## Abstract

More than a year and a half has passed since the novel coronavirus (COVID-19) pandemic began, but according to the World Health Organization (WHO), the end is still a long way off. People must incorporate infection prevention behaviors into their daily lives, and the question for the future will not be whether or not to take countermeasures but how thoroughly to do so. In this study, I conducted an online survey of 1200 Japanese adults and identified four patterns of implementation of preventive measures. Those who took the most preventive measures were highly alert to the risk of COVID-19 and had strong anxiety about infection. They also positively evaluated risk management by medical institutions and the government, and they had positive feelings in their daily lives. On the other hand, those who took minimal measures, mainly mask wearing and handwashing, and those who took less than minimal measures did not feel much risk or anxiety about COVID-19. However, they evaluated the responses of the government and medical institutions less highly and reported having more negative feelings in their daily lives.

## 1. Introduction

**Figure 1:**
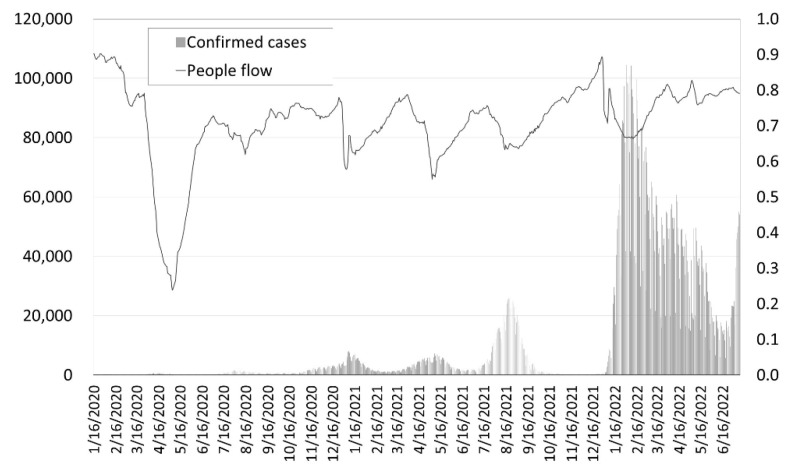
Number of confirmed cases in Japan.

In December 2019, a novel coronavirus (COVID-19) infection was confirmed in Wuhan, Hubei Province, People’s Republic of China, and World Health Organization (WHO) was declared a pandemic on 11 March 2020. As of 5 July 2022, 547,901,157 people worldwide had been confirmed infected (including 6,339,899 deaths) [1], and 5,239,721,051 people (67.2% of the world population) had received at least one dose of vaccine [2]. In Japan, the cumulative numbers of positive cases and deaths as of 10 July 2022 were 9,676,600 and 31,418, respectively [3]. Figure 1 was created by the author from the published data of the Cabinet secretariat. The gray vertical bars in Figure 1 refer to the number of confirmed cases (left axis). As you can see from the figure, Japan has experienced six peaks in the spread of the disease so far.

Japan’s COVID-19 Countermeasures Promotion Office, Cabinet Secretariat, calls for the implementation of five countermeasures to prevent the spread of infection: avoiding dense, close, and closed rooms; keeping a safe distance from others; washing hands frequently; room ventilation and cough etiquette; and installing contact confirmation applications on smartphones [4]. As for the public’s countermeasure actions, the percentage of people who wear masks in public places has remained high for two years: 62% in March 2020, 86% in May, and 89% in December, 89% in April 2021, 88% in December, and 87% in April 2022 [5]. The movement of people has also been largely suppressed, with the number of people in entertainment areas in particular continuing to decline significantly compared to pre-pandemic levels [6]. The broken line in Figure 1 shows the flow of people into the main downtown area at 3 p.m. If 2019, the year before the coronavirus pandemic, is set at 1.0 (right axis), it is below 1 for the entire period.

To control the spread of infection, it is important for each individual to implement preventive measures, and many previous studies have investigated which factors contribute to the people’s compliance with preventive measures for infectious diseases, including COVID-19. For instance, the perception of one’s risk of infection or severe illness contributes to mask use [7,8]. Moreover, attitudes toward social norms and adherence to norms contribute to mask use and compliance with WHO’s guidelines for preventive behavior [9,10]. The perceived effectiveness of countermeasure behaviors and perceived risk also contribute to mask wearing, handwashing and disinfection, and social distancing [11,12]. In addition, anxiety about risk and the anticipation of regret influence behavioral choices [13], and self-efficacy influences behavior, including the choice to act [14,15]. Synchrony contributes to handwashing behavior, while risk perception has no effect on preventive behavior in these studies [16]. In the author’s study, the effectiveness of measures, altruism, and vigilance increased the number of preventive measures taken, but risk perception had no effect [17]. In other words, various factors, such as risk perception and attitude toward COVID-19, as well as emotions such as anxiety and regret, normative consciousness, and sympathy influence the implementation of infection prevention measures.

People have been living with such preventive measures for two years, and they will continue to have to adjust their preventive actions according to the status of COVID-19 infection. Preventive measures include not only wearing masks and washing hands, but also avoiding crowded places, refraining from going out, ventilating rooms, using outdoor air-conditioning, maintaining social distance, and taking care of one’s own health. As the pandemic has become prolonged, the question is not whether or not to take preventive measures but how thoroughly to implement them based on the infection situation in society and one’s own lifestyle.

This study aims to clarify how people implement and combine preventive measures for COVID-19 and the reasons behind their choices. I will also clarify the patterns of people’s infection prevention behavior as well as their perceptions and attitudes toward COVID-19, with the aim of gaining knowledge that will contribute to the promotion of effective preventive actions by public health administrations.

## 2. Materials and Methods

### 2.1. Procedures

A cross-sectional online survey was conducted with a nationally representative Japanese sample (1200) of 1200 respondents, who were chosen based on Japanese demographics: prefecture of residence, gender, and age (18 years and older in 10-year increments). The construction of the online survey platform, sending of cooperation request emails to survey monitors, reminder, and exclusion of missing data were handled by a private research company, Cross Marketing Inc. (Tokyo, Japan).

### 2.2. Questionnaire

The questionnaire consisted of items about COVID-19 risk characteristics (16 items, 7-point semantic differential scale), anxiety (15 items, 5-point scale), infection preventive behaviors (16 items, multiple selection), reasons for these behaviors (13 items, multiple selection), recognition (11 items, 5-point scale), evaluation of government or institutional responses toward the pandemic (3 actors, 5-point scale), evaluation of ability and commitment in responses toward the pandemic (3 actors, 5-point scale), and feelings in daily life (7 items, 7-point semantic differential scale).

The survey was approved by the Ethics Review Committee of the university with which the author is affiliated (approval no. 21-10).

It was conducted over a six-day period from 5–10 November 2021. This is the timing of the convergence of the fifth wave of new coronavirus infections (see Figure 1).

All the study procedures are carried out as per principles described in the declaration of Helsinki.

### 2.3. Analysis

The sample was subjected to descriptive analysis using percentages for qualitative variables and means for quantitative variables. The statistical software used was JMP 13 (SAS Institute, NC, USA). Factor analysis was performed for the quantitative variables risk characteristics, anxiety, and recognition; two factors were extracted for COVID-19 characteristics through factor analysis (varimax rotation). Moreover, two factors were extracted for anxiety and recognition toward COVID-19 each. Cluster analysis (k-means method) was used to extract patterns of implementation of preventive measures, and four clusters with different numbers of implemented measures were extracted. The Cube Clustering criterion (CCC) is used in SAS as an indicator of the number of clusters to be extracted [18]. The number of clusters with a CCC greater than 3 and a larger value is estimated to be optimal, thereby suggesting that 6 is the optimal number in this analysis (CCC = 21.6). However, with clusters numbering 6, the average number of implemented measures is 1.6, 7.8, 9.6, 10.4, 9.3, and 3.3, which cannot be a clustering that corresponds to the purpose of extracting clusters with different degrees of thoroughness in implementing many measures. Therefore, the number of clusters was set to 4 (CCC = 8.6), wherein the number of countermeasures implemented in each cluster is discriminable and CCC > 3 is satisfied.

Multiple regression analysis was conducted for each cluster to examine the influence of each factor on the implementation of preventive actions. A one-way analysis of variance was also conducted using the mean of the ratings of government and agency officials on their response to the pandemic, and groups with different patterns of implementation of preventive measures were compared.

## 3. Results and Discussion

The following results are based on an analysis of 579 men (48.3%) and 621 women (51.8%) with a mean age of 52.2 years (range: 18–91).

### 3.1. Perception of COVID-19

#### 3.1.1. Perception COVID-19 Risk Characteristics

A factor analysis (varimax rotation) was performed on the data of all participants to determine how they perceived the risks of COVID-19, using 14 items, and two factors were extracted (Table 1). The first factor, called “difficulty in responding”, consists of four items: “threat to future generations (How great a threat to future generations will the damage be?),” “ease of reduction (How easy is it to reduce risk?),” “catastrophe (Will one person die at a time from this or will many people simultaneously lose their lives?),” and “lethality (How likely is it to lead to death?).” The second factor, labeled “trivial”, consists of two items: “old (Is it new or old and familiar?)” and “few people exposure (How many people are exposed to it?).”

#### 3.1.2. Anxiety

The participants were asked about the degree to which their concerns about COVID-19 applied to 15 statements, with responses given on a 5-point scale. Factor analysis was conducted, and two factors were extracted (Table 2). The first factor consisted of five items: “personally being infected with COVID-19,” “pressure on medical institutions,” “not knowing when the infection will subside,” “infecting others,” and “time needed to develop and approve vaccines and medical treatments.” Because these items can be regarded as concerns related to COVID-19 infection, they were named the “infection anxiety” factor.

The second factor consisted of five items: “changes in working and learning styles due to telework, online classes, etc.,” “deterioration and diminishing of personal relationships due to lack of communication with friends and colleagues,” “education and exams for myself and my children,” and “stress caused by staying at home for long hours.” This was termed the “social life anxiety” factor because the items concern anxiety about the effects of measures taken to restrict social life in order to prevent the spread of the COVID-19 infection.

#### 3.1.3. Recognition to COVID-19

The factor analysis using the degree of agreement with 11 descriptions regarding perceptions of COVID-19 revealed two factors. As shown in Table 3, the first factor, designated the “alert” factor, consists of five items: “COVID-19 is an issue that transcends generations, regions, and countries,” “COVID-19 is close at hand,” “COVID-19 is an important issue for me personally,” “Infection can happen to anyone, no matter how many measures are taken,” and “To control COVID-19, no matter what my personal situation is, I must refrain from going out unnecessarily and avoid the Three Cs (closed spaces, crowded places, and close-contact settings).” 

The second factor, called the “omission factor,” consists of the following five items: “Individual behavior will not make any difference in controlling COVID-19,” “I do not want to change my behavior to prevent COVID-19,” “The extent of the socioeconomic crisis caused by COVID-19 is exaggerated,” “Individuals should not change their lives to a large extent,” and “New technologies will solve COVID-19 without major changes in individuals’ lives.”

Table 4 shows the results of correlation analysis among independent variables for multiple regression excluding dummy variables. To avoid multicollinearity, difficulty in responding, social life anxiety, and alert are excluded from the multiple regression analysis. Table 5 shows the results of multiple regression analysis with the number of measures implemented (maximum 16; see Table 6 for breakdown) as the dependent variable. The independent variables are risk characteristics, anxiety, recognition, and reasons (see Table 7). The mean number of practices was 6.15, with 37% of the variation explained by the variables in the model. The higher the anxiety about infection, the higher the number of countermeasures implemented. The stronger the perception of COVID-19 as a trivial risk and the perception of inaction (e.g., that one’s actions do not contribute to reducing the spread of infection and that the effects of COVID-19 are exaggerated), the lower the number of countermeasures implemented. The effectiveness of countermeasures, for example, altruism about not wanting to infect others, perception of one’s risk of serious illness, and not wanting to experience regret after infection, significantly promoted the implementation of the countermeasures. The number of measures taken by women was higher than that by men, and the number of measures taken increased with age.

The effects of anxiety, awareness of COVID-19, perceived effectiveness of measures, perceived risk, and emotions toward the implementation of infection prevention measures are consistent with previous studies. However, while previous studies [8,9,19] on emerging infectious diseases have shown that concurrence has a significant effect on whether a person wears a mask, concurrence alone did not have a significant effect among the independent variables entered in this study. This indicates that while conformity to the behavior of those around one contributes to the decision whether to act, conformity may not contribute to the extent that multiple measures are implemented.

### 3.2. Patterns of Implementation of Preventive Measures for COVID-19

To determine the extent to which people are thoroughly implementing countermeasure actions as well as the characteristics and number of combinations of countermeasure actions, a cluster analysis was conducted on the implementation of the 16 preventive measures presented in the questionnaire. As a result, four clusters were extracted (Table 6). Cluster 3 was the largest, accounting for about 45% of all participants with an average of 3.4 preventive measures implemented. Cluster 4 was the second largest with an average of 7.8 preventive measures. Cluster 1 comprised about 18% of the participants, with an average of 11.0 preventive measures. Cluster 2, the smallest, included about 4% of the participants and had the lowest average number of preventive measures implemented at 1.2.

The table shows which specific preventive measures were practiced. The measures implemented by 50% of the participants in each cluster are hatched. In Cluster 1, more than half of the participants took 13 measures except “wearing gloves” and “PCR test,” whereas in Cluster 2, there were no preventive measures taken by the majority of participants, indicating that the preventive actions taken by each individual varied. Based on the patterns of implementation of preventive actions, I refer to Cluster 1 as the Maximum preventive group, Cluster 2 as the No-preventive group, Cluster 3 as the Minimum group, and Cluster 4 as the Intermediate group. 

The vaccination rate was 86.3%, 84.4%, and 77.2% in the Intermediate, Maximum, and Minimum groups, respectively, and 43.2% in the No-preventive group. The proportion of participants who reported that they had not received any vaccination was 4.7% in the Maximum group, 4.4% in the Intermediate group, 8.4% in the Minimum group, and 29.6% in the No-preventive group.

**Table 6:** Ratio of the implementation of preventive measures.

	Maximum	Intermediate	Minimum	No-Preventive
Number of observations	211 (17.6)	410 (34.2)	535 (44.6)	44 (3.7)
Mean of implementation of preventive measures	11.0	7.8	3.4	1.2
Use of mask	100.0	100.0	100.0	0.0
Infection control measures such as hand washing or cleaning fingers using an antiseptic solution	99.1	95.9	80.4	31.8
Use of gloves	23.7	7.8	7.1	6.8
Use of cashless payments	70.6	46.3	24.3	11.4
Refraining from leaving home for nonurgent or nonessential purposes	90.1	83.2	30.3	9.1
Postponing or canceling travel or leisure activities	91.0	75.6	16.3	9.1
Refraining from eating out	85.8	80.2	20.2	11.4
Having sufficient exercise, nourishment, and sleep	70.6	49.0	21.3	4.6
Refraining from experiential entertainment	80.1	44.9	1.9	9.1
Refraining from physical contact, including handshakes and hugs	89.1	55.4	11.0	4.6
Moving by car or bicycle rather than public transportation	69.7	44.9	6.5	2.3
Communication using online tools rather than face-to-face	62.6	3.7	2.2	2.3
Use of mail order and delivery services	62.6	4.6	1.9	2.3
Avoiding crowded locations and times to the extent possible	88.2	84.6	14.6	6.8
Getting a PCR test	13.7	4.2	1.9	4.6
Nothing	2.4	1.7	0.4	2.3

Table 7 shows the reasons for implementing or not implementing preventive measures by group. The most frequently selected reasons for taking action in the Maximum group were the following: “I think it is effective in preventing infection [effectiveness]” (89.1%), “I don’t want to infect other people [altruism]” (83.4%), and “I don’t know if it has any effect, but I don’t want to regret not doing it [regret]” (61.6%), in that order. The Intermediate group also had a high selection rate for the same order of reasons for implementing measures. The Minimum group selected effectiveness (73.8%) and altruism (52.5%) as the top two reasons, like the other groups, but the third most frequently selected reason was “Everyone around me is doing it [conformity]” (23.0%), indicating a strong tendency to take action in accordance with those around them rather than because they did not want to regret their actions. More than 20% of the participants in both the Maximum (29.9%) and Intermediate (23.4%) groups reported conformity as a motivation, suggesting that the reason people take countermeasures includes a sense of peer pressure. On the other hand, the reason selected most frequently by the No-preventive group was “I don’t think it would be a big deal if I were infected” (31.8%).

**Table 7:** Reasons for implementing/not implementing preventive measures.

	Maximum	Intermediate	Minimum	No-Preventive
I think it is effective in preventing infection (effectiveness)	89.1	89.0	73.8	27.3
I don’t want to infect other people (altruism)	83.4	79.5	52.5	25.0
I am at high risk of aggravation (risk perception)	46.0	34.2	17.6	6.8
I live with someone who is at high risk of serious illness	17.5	14.9	7.1	4.6
I don’t know if it has any effect, but I don’t want to regret not doing it (regret)	61.6	50.7	15.9	6.8
Everyone around me is doing it (conformity)	29.9	23.4	23.0	9.1
The procedures and preparations for countermeasures are troublesome	3.8	2.4	1.3	0.0
It is costly	3.3	3.9	4.3	13.6
I do not know what specific measures to take	5.7	3.4	2.1	6.8
I have doubts about how effective the countermeasures will be	8.1	5.9	4.7	15.9
There are few infected people in my area	8.1	5.9	6.0	6.8
I don’t think it would be a big deal if I were infected	1.9	1.0	4.5	31.8

The mean age, gender ratio, and risk perceptions and recognitions concerning COVID-19 for each group are shown in Table 8. For perceptions and recognitions, I used the average of the responses to the questions comprising each of the aforementioned factors. All responses were given on a 5-point scale, with higher scores indicating a stronger tendency toward that factor.

The mean age of the Maximum and Intermediate groups was 57.2 years old, the highest among the four groups, and both groups had more women than men. The No-preventive group had the lowest mean age and the highest percentage of men. The perception of COVID-19 as a difficulty in responding was highest in the Maximum and Intermediate groups, followed by the Minimum and No prevention groups, with a significant difference in mean values (*F*(3,1196) = 37.0, *p* < 0.001). Conversely, the means of perceiving the risk as trivial was highest in the No prevention group, followed by the Minimum, Intermediate, and Maximum groups (*F*(3,1196) = 12.5, *p* < 0.001). Infection and social life anxiety were highest in the Maximum and Intermediate groups, followed by the Minimum group, and lowest in the No prevention group (infection anxiety; *F*(3,1196) = 65.4, *p* < 0.001; social life anxiety; *F*(3,1196) = 9.2, *p* < 0.001). For alertness to COVID-19, the Maximum and Intermediate groups had the highest mean scores, followed by the Minimum and No-preventive groups (*F*(3,1196) = 92.1, *p* < 0.001). However, the mean score for omission was highest in the Minimum group and lowest in the Maximum and Intermediate groups (*F*(3,1196) = 25.0, *p* < 0.001).

Thus, about half of the Japanese population took only minimal measures, and the other half took an average of seven or more measures in combination, of which 18% took an average of 11 measures in combination. The Maximum and Intermediate groups, who implemented greater combinations of measures, perceived COVID-19 as a major threat and were concerned about infection. They believed that the countermeasures were effective, did not want to infect others with COVID-19, and did not want to regret infecting themselves without taking countermeasures; therefore, they took actions such as wearing masks, disinfecting their hands, reducing face-to-face contact with others, and avoiding going out and visiting crowds. The Minimum group, who took only minimal measures such as wearing masks and sanitizing their hands, recognized COVID-19 as a difficult risk, but were less concerned about infection and less conscious of their own risk of becoming seriously ill, and of not wanting to pass it on to others. The No-prevention group was even less anxious and wary of infection than the Minimum group, and was most likely to perceive COVID-19 as a trivial problem and an exaggerated risk. The No-prevention group was more likely to be optimistic about COVID-19 and underestimate the risk, thereby indicating that they did not believe that action was necessary to prevent COVID-19.

### 3.3. Differences in the Perception of the Current Situation across Groups

Table 9 shows the results of a 5-point scale for the three actors (national government, medical institutions, and individuals) in terms of how the participants perceived the current situation of the pandemic, and whether the actors were responding well, had the ability to respond appropriately, and were taking the situation seriously. On the scale, 3 points means “can’t say either way,” and a score higher than 3 indicates a positive evaluation.

All groups assigned low ratings to both the government’s response and its ability to respond. In contrast, medical institutions were highly evaluated for their response as well as for their ability and serious commitment in dealing with COVID-19, while individuals were also evaluated as taking the problem seriously and dealing with it well. The average rating given by the No-preventive group was below 3, not only for the government administration, but also for medical institutions and individuals, indicating that the participants in this group believed there was a poor response, lack of ability to respond, and lack of serious efforts.

The questionnaire also asked about the participants’ expectations for the national and prefectural governments. The participants were given 13 answer choices, and the average number of response items chosen was 5.5 were for the Maximum group, 4.6 for the Intermediate group, 2.4 for the Minimum group, and 1.7 for the No-preventive group. In addition, 31.8% of the participants in the No-preventive chose the exclusive option, “I do not expect anything from the government.” In other words, the participants who took many preventive measures against COVID-19 tended to give a higher evaluation of and expect more from administrative agencies, while those who did not take preventive actions had a lower evaluation of and expected less from administrative agencies’ ability to respond.

The Minimum and No-prevention groups, who were less anxious and wary about COVID-19, more strongly perceived the national government and medical institutions’ response to be inadequate than the Maximum and Intermediate groups, thereby suggesting that they were not optimistic about the current status of the pandemic as a social situation, even if they considered measures against the risk personally unnecessary.

**Figure 2:**
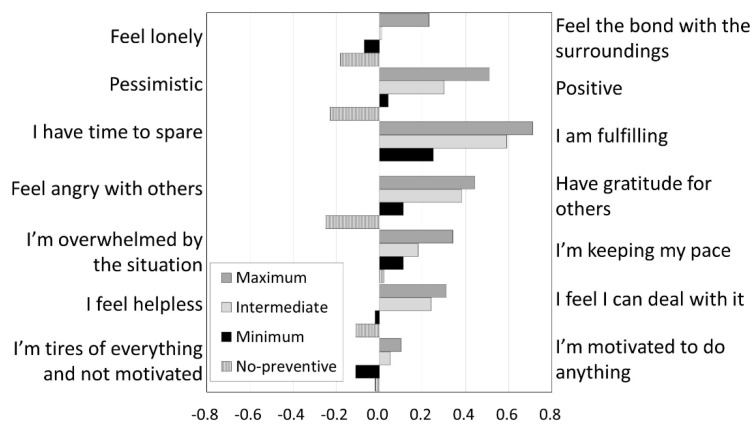
Feelings in daily life.

The results of the items about feelings in daily life are shown in Figure 2. The responses measured by the semantic differential method of the 5-point scale with the feelings in the figure placed on both sides were averaged for the groups, with “neither” (middle response option) set at 0, agreement with negative feelings on the left side, and agreement with positive feelings on the right side. As the figure shows, there was a positive tendency in the Maximum and Intermediate groups. In comparison, the No-preventive group had a low reporting of positive feelings, and the Minimum group had a tendency toward negative feelings.

## 4. Conclusions

Consistent with previous studies, this study showed that the perceived effectiveness of the measures, altruism of not wanting to transfer the disease to others, and feeling of not wanting to regret it, along with anxiety and caution, had a significant influence on the implementation of additional COVID-19 preventive measures. I extracted four implementation patterns from the number of times COVID-19 prevention measures were implemented and clarified the characteristics of people’s risk perception, awareness, and anxiety in each behavioral pattern. Those who took more frequent measures were more alert and anxious about COVID-19, recognized their own risk of serious illness and the effectiveness of the measures, and acted to avoid infecting others. Conversely, those who took only two or three minimal countermeasures were less anxious and worried about COVID-19, estimated their own risk of severe infection to be low, and did not feel the need to take countermeasures. However, those who did not take countermeasures valuated the adequacy and competence of the administration and medical institutions’ response less favorably. The relationship between the government and individual countermeasures is not complementary: individuals take many countermeasures for self-protection because the government’s countermeasures are insufficient, or they are optimistic about COVID-19 because the government and medical institutions are functioning adequately and are not taking countermeasures themselves, but a positive relationship exists between the adequacy of individual countermeasures and those of the government and medical institutions. The results showed a positive relationship between the evaluation of the response of the government and medical institutions. Those who implement an average combination of 7–11 measures in their daily lives are concerned and worried about COVID-19 without being frightened of the risk and driven by the measures, but they evaluate the responses of the government and medical institutions positively and live their daily lives with positive emotions. However those who evaluate the risk of COVID-19 as small and do not act in a self-protective manner are more likely to evaluate the response of society negatively and live with negative emotions.

Until COVID-19 is treated like a seasonal influenza endemic, people will continue to have to integrate infection prevention into their daily lives. How thoroughly infection prevention is implemented will depend on individual decisions based on personal risk, health status, and the infection situation in society. Public health administrations responsible for risk management should continue to provide information not only on the health risks of COVID-19 and the current infection spread status, but also on why thorough measures are necessary and how effective they are expected to be, depending on the situation, in order to support individual decision-making. As previously mentioned, multiple factors determine preventive behavior, and effective factors are likely to differ depending on individual attributes such as gender, age, risk of serious illness, health status, family structure, and lifestyle patterns. Rather than a one-size-fits-all message, it is necessary to determine a specific target audience and tune the message according to their characteristics.

Although this research only reveals a correlation between not actively taking preventive measures and negative feelings in daily life and low evaluations and expectations of government agencies, it is possible that some kind of mental disorder caused by the living environment during the pandemic may be involved in both. Alternatively, it is possible that taking all possible preventive measures has a positive effect on the sense of efficacy in daily life. Several previous studies have shown that pandemics impose significant constraints on people’s social lives, with undesirable economic and psychological effects [20,21]. Furthermore, low trust in institutional responses to pandemics is correlated with feelings of anxiety and lack of control [22]. Additionally, individual differences have been shown in vulnerability to changes in the living environment, such as lockdowns, due to psychological and economic circumstances, such as anxiety and depression [23,24]. Although mandatory lockdowns have not been implemented in Japan, they have affected the mental health of college students through the burden of distance learning and its economic impact [25]. These findings suggest that a sense of mental burden may lead to a decrease in countermeasure behavior, because mental health and spare time availability make many countermeasure behaviors possible, and it takes time and effort to thoroughly implement countermeasure behaviors.

The results of this study suggest that in considering public health policies and risk communication to support individual decisions to take thorough preventive measures, it is important to focus on the direct health and economic and social effects of COVID-19, and consider the emotional and mental health of those who have not taken preventive actions.

## Figures and Tables

**Table 1:** Results of the factor analysis of risk characteristics.

	Difficulty in Responding	Trivial
How great a threat to future generations will the damage be?	0.74	−0.08
How easy is it to reduce risk?	0.71	−0.11
How likely is it to lead to death?	0.68	0.06
Will one person die at a time from this, or will many people simultaneously lose their lives?	0.70	0.065
Is it new or old and familiar?	0.17	0.64
How many people are exposed to it?	−0.20	0.61

**Table 2:** Results of factor analysis regarding anxiety.

	Infection Anxiety	Social Life Anxiety
Personally being infected with COVID-19	0.79	0.24
Pressure on medical institutions	0.79	0.21
Not knowing when the infection will subside	0.78	0.23
Infecting others around me	0.75	0.29
Time needed to develop and approve vaccines and medical treatments	0.62	0.35
Changes in working and learning styles due to telework, online classes, etc.	0.22	0.70
Deterioration and diminishing of personal relationships due to a lack of communication with friends and colleagues	0.31	0.68
Education and exams for myself and my children	0.14	0.64
Stress caused by staying at home for hours	0.30	0.62

**Table 3:** Results of factor analysis of COVID-19 recognition.

	Alert	Omission
COVID-19 is an issue that transcends generations, regions, and countries.	0.80	−0.15
COVID-19 is close at hand.	0.76	−0.10
COVID-19 is an important issue for me personally.	0.73	−0.10
Infection can happen to anyone, no matter how many measures are taken.	0.66	0.02
Regardless of one’s personal circumstances, to limit COVID-19, one should not leave home for nonessential and nonurgent reasons and should avoid the three C’s, etc.	0.60	0.03
I do not want to change my personal behavior to prevent COVID-19.	−0.13	0.66
Individual behavior changes nothing to control COVID-19.	−0.15	0.68
The scope of the socioeconomic crisis posed by COVID-19 is exaggerated.	−0.01	0.59
COVID-19 will be resolved using new technologies without individuals making major lifestyle changes.	0.16	0.51

**Table 4:** Correlation between cognitive variables.

	Difficulty in Responding	Trivial	Infection	Social Life	Alert
Risk characteristics: Trivial factor	−0.03				
Anxiety: Infection factor	0.45	−0.23			
Anxiety: Social life factor	0.19	0.11	0.53		
Recognition: Alert factor	0.48	−0.30	0.58	0.20	
Recognition: Omission factor	−0.15	0.31	−0.10	0.20	−0.10

**Table 5:** Main factors that determine the number of implementation measures.

Variables	*β*	*p*
Risk characteristics: Trivial factor	−0.15	*
Anxiety: Infection anxiety factor	0.62	***
Recognition: Omission factor	−0.55	***
Reason: Effectiveness	0.29	**
Reason: Altruism	0.52	***
Reason: Risk perception	0.39	***
Reason: Regret	0.94	***
Reason: Conformity	0.03	
Gender	0.31	***
Age	0.03	***
Partial	4.29	***
Adjusted R^2^	0.37	***
*n*	1200	
Dependent variable: No. of implemented preventive behaviors	6.15	

(Reason) Dummy variable (Selected = 1, Not selected = 0); (Gender) Dummy variable (Male = 1, Female = 2); * *p* < 0.05 ** *p* < 0.01 *** *p* < 0.001.

**Table 8:** Characteristics of the four preventive groups (clusters).

	Maximum	Intermediate	Minimum	No-Preventive
Age	57.2	57.2	47.0	45.6
Gender	42.7/57.4	38.3/61.7	56.1/43.9	74.7/27.3
Risk characteristics: Difficulty in responding	4.67	4.72	4.19	3.68
Risk characteristics: Trivial factor	2.98	3.01	3.41	3.57
Anxiety: Infection factor	3.92	3.91	3.39	2.69
Anxiety: Social life factor	3.13	3.06	2.93	2.51
Recognition: Alert factor	4.05	3.95	3.46	2.83
Recognition: Omission factor	2.53	2.58	2.90	2.70

**Table 9:** Evaluation of the COVID-19 risk management of each actor.

	Maximum	Intermediate	Minimum	No-Preventive
Responded well/National government	2.65	2.56	2.61	2.05
/Medical institutions	3.58	3.60	3.21	2.55
/Individuals	3.20	3.21	3.03	2.55
Ability of appropriate response/National government	2.63	2.50	2.54	2.09
/Medical institutions	3.70	3.65	3.20	2.59
/Individuals	3.06	3.06	2.84	2.43
Serious commitment to response/National government	3.22	3.04	2.96	2.20
/Medical institutions	4.04	4.06	3.52	2.80
/Individuals	3.49	3.44	3.13	2.80

## Data Availability

The datasets generated during and/or analyzed during the current study are available from the corresponding author on reasonable request.
